# HIGH RESOLUTION MASS SPECTROMETRY IN LIPIDOMICS

**DOI:** 10.1002/mas.21627

**Published:** 2020-03-31

**Authors:** Thomas Züllig, Harald C. Köfeler

**Affiliations:** ^1^ Core Facility Mass Spectrometry Medical University of Graz, ZMF Graz Austria

**Keywords:** lipidomics, mass spectrometry, chromatography, high mass resolution

## Abstract

The boost of research output in lipidomics during the last decade is tightly linked to improved instrumentation in mass spectrometry. Associated with this trend is the shift from low resolution—toward high‐resolution lipidomics platforms. This review article summarizes the state of the art in the lipidomics field with a particular focus on the merits of high mass resolution. Following some theoretical considerations on the benefits of high mass resolution in lipidomics, it starts with a historical perspective on lipid analysis by sector instruments and moves further to today's instrumental approaches, including shotgun lipidomics, liquid chromatography–mass spectrometry, matrix‐assisted laser desorption ionization‐time‐of‐flight, and imaging lipidomics. Subsequently, several data processing and data analysis software packages are critically evaluated with all their pros and cons. Finally, this article emphasizes the importance and necessity of quality standards as the field evolves from its pioneering phase into a mature and robust omics technology and lists various initiatives for improving the applicability of lipidomics. © 2020 The Authors. *Mass Spectrometry Reviews* published by John Wiley & Sons Ltd. Mass Spec Rev

## I INTRODUCTION

Lipids are one of the major compound classes in biological systems and fulfill important physiological tasks. Their hydrophobicity enables them to form cellular membranes that constitute a boundary against the cells hydrophilic surroundings. This compartmentalization is the physical basis of any living entity. The second important biological task of lipids is energy storage. Lipids are perfectly suited for this physiological duty due to the high amount of energy generated by their oxidation. The third task fulfilled by lipids is signaling, by participation in intermolecular and intramolecular autocrine, paracrine, and endocrine regulatory processes.

**Figure 1:**
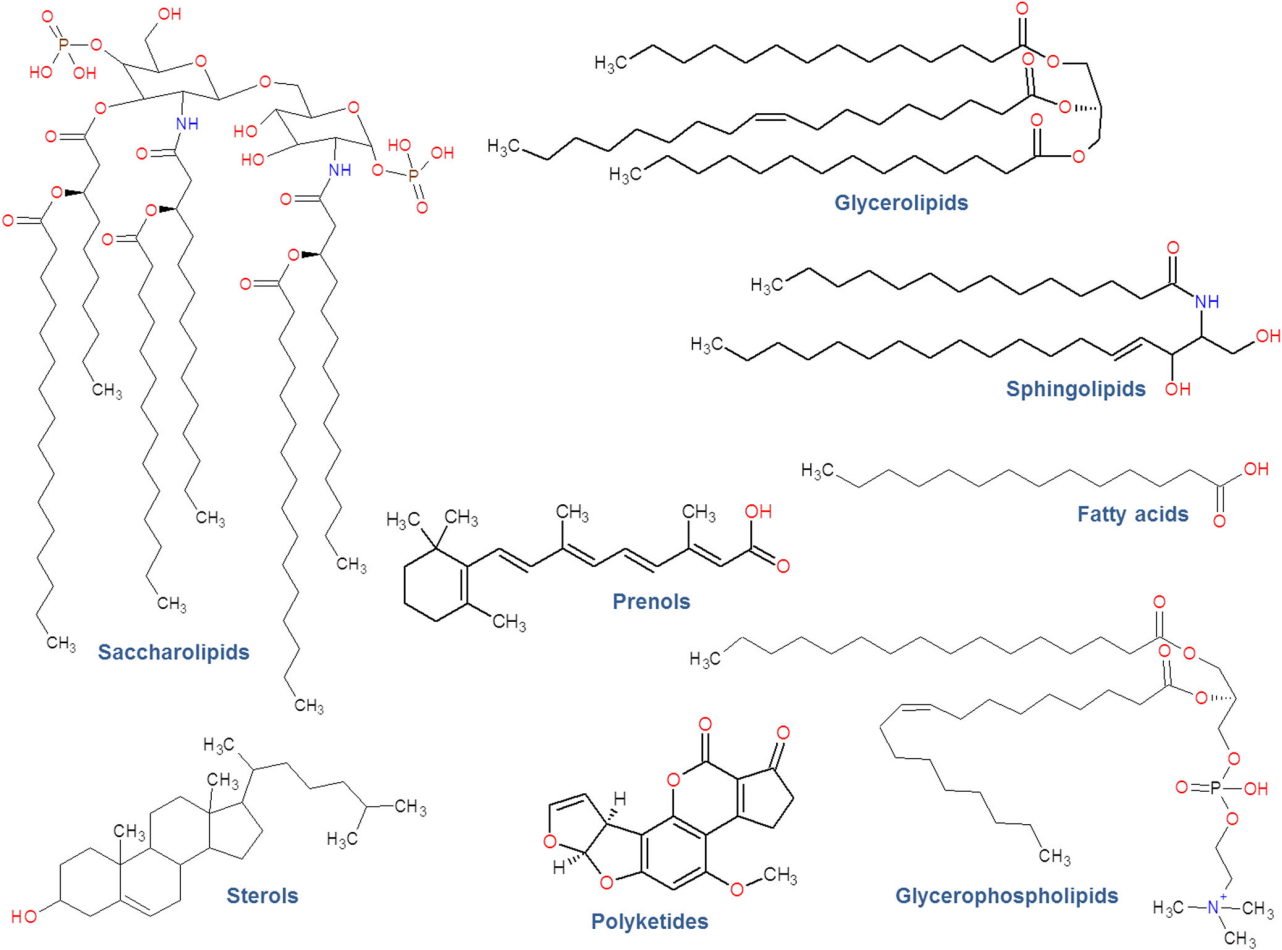
Example structures for the eight lipid categories as defined by the International Lipids Classification and Nomenclature Committee (ILCNC) in 2005 and implemented in the LIPID MAPS Structure Database (LMSD). [Color figure can be viewed at wileyonlinelibrary.com]

Besides their biological functions lipids are classified into eight categories according to their chemical building blocks (1) fatty acyls, (2) glycerolipids, (3) glycerophospholipids, (4) sphingolipids, (5) sterols, (6) prenol lipids, (7) saccharolipids, and (8) polyketides are all based on fatty acyls/fatty alkyls, sphingosine, or prenol as basic hydrophobic building blocks (Fahy et al., 2005) (Fig. 1). All categories are further subdivided into lipid classes and lipid subclasses. In total 43,659 individual lipids—21,706 curated compounds and 21,953 computationally generated compounds—are compiled in the LIPID MAPS structure database (LMSD), which is the most comprehensive database in the field of lipidomics. Nevertheless, it is speculated that the number of naturally occurring lipids ranges at 100,000 or even more species, which is still far from the numbers annotated in databases.

As stated above, lipids play an important role in many cellular processes and hence are also involved in formation, prolongation but also resolution of many diseases like chronic inflammation, cardiovascular and neurodegenerative disorders, diabetes, or cancer to mention just the most representative ones. Thus, identification and also subsequent quantification of lipids has become an important need in biomedical research and the most suitable method for archiving this goal is clearly mass spectrometry (Rustam & Reid, 2018). This is reflected in a tremendous increase of the publication output in the last 10 years. According to Web of Knowledge the number of publications for the search term “Lipidomics” increased by a factor of 7.7 during this period, which makes it one of today's fastest‐growing research fields. One of the reasons for this astonishing success is the availability of constantly developing chromatographic, mass spectrometric, and bioinformatics tools alike. Particularly high‐resolution mass spectrometry had a big impact on this success story in the last decade. This review article will, therefore, put its emphasis on the development and use of high‐resolution methods for lipidomic analysis.

## II THEORETICAL CONSIDERATIONS

**Figure 2:**
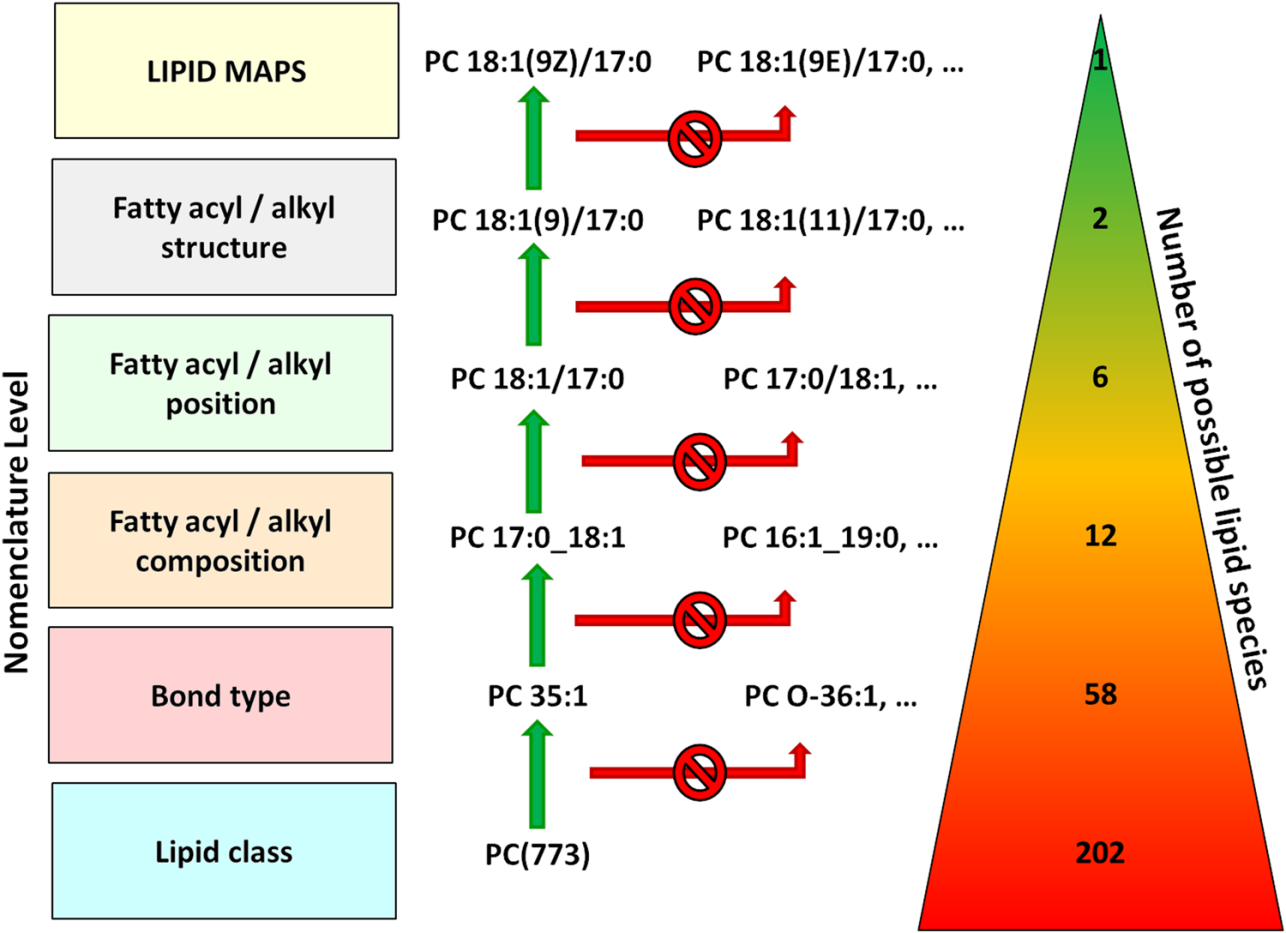
The possible number of molecular PC structures at *m*/*z* 773 shrinks with higher levels of structural elucidation until finally only one possibility remains. Concomitantly the compound annotation reflects the level of structural depth according to the lipid shorthand nomenclature. [Color figure can be viewed at wileyonlinelibrary.com]

One major challenge in lipidomics is the extremely high diversity of molecular lipid species to be expected in most biological samples, which, in turn, is to a high degree a result of the combinatorial possibilities arising from combination of the various building blocks of lipids. This fact leads to many potential mass spectral overlaps of lipid molecular ions and molecular adduct ions. While isomeric species do have exactly the same elemental composition and cannot be separated by mass spectrometry without fragmentation, isobaric lipids do not have the same elemental composition and can thus be separated with sufficiently high mass resolution. Table 1 lists some of the most commonly encountered isobaric overlaps in lipidomics. The resolution values listed in the table can be considered as rough approximations for the mass width needed at half the height of the peak at the respective *m*/*z* values in order to achieve full baseline separation. Full baseline separation gets particularly important when an isobaric species at low mass spectral intensity is to be observed beside, for example, a highly abundant major lipid species. The more the peak intensities of both isobars are starting to equalize the less resolution is needed, because clear baseline separation is becoming less and less important for separating isobaric *m*/*z* values of about equal height, when compared with isobaric *m*/*z* values with larger inequalities in peak height. The first four examples of the table can routinely be separated by quadrupole time‐of‐flight (Q‐TOF) technology, which for some instruments even reaches up to 80,000 resolution. While the first example depicts a highly unsaturated phosphatidylethanolamine species besides a saturated phosphatidylcholine (PC) species (^12^C_1_ vs. ^1^H_12_) the second example shows the often observed overlap of PC and phosphatidylserine species (^12^C_2_^1^H_8_ vs. ^16^O_2_), which is basically due to a two oxygen difference in elemental composition. Both examples depict monoisotopic peaks of protonated adducts commonly detected in lipid mass spectra. The mass difference between plasmalogens and odd fatty acyl carbon numbered diacyl species shows only one oxygen difference in elemental composition (^16^O_1_ vs. ^12^C_1_^1^H_4_) and is, therefore, harder to detect than the previously mentioned two oxygen “shift”. Another issue often encountered are overlaps of monoisotopic and *M* + 1 peaks between even and odd mass ions, which is particularly true for sphingomyelin and PC species (^13^C_1_^1^H_5_^14^N_1_ vs. ^16^O_2_). This is usually just observed in shotgun lipidomics without any chromatographic pre‐separation and requires about 30,000 resolution. The by far most widely observed isobaric overlap in lipidomics is between the monoisotopic peak of a lipid species and the *M* + 2 peak of the same lipid species with just one additional double bond in its fatty acyl chains (^12^C_2_^1^H_2_ vs. ^13^C_2_). The resolution needed for resolving these overlaps is roughly 180,000, dependent in its exact number on the mass and the intensity relation of both peaks. This resolution is only achievable by Fourier transform‐ion cyclotron resonance‐mass spectrometry (FT‐ICR‐MS) or by certain Orbitrap instruments. When such *M* + 2 or also *M* + 1 peaks are not resolved either in their *m*/*z* dimension or in their chromatographic dimension isotopic correction functions are needed for calculation of monoisotopic peak intensities (Han & Gross, 2005). In a nutshell, by knowledge of the natural abundance of ^13^C and the intensity pattern of the peak cluster under investigation, it is possible to calculate the percentage contribution of monoisotopic and *M* + 1 or *M* + 2 masses, even when they are not mass resolved. According to the concept proposed by Wang, Huang, and Han (2014) even a mass resolution of about 75,000 at *m*/*z* 750 would be sufficient for separately identifying and quantifying both species. At this mass resolution the *M* + 2 peak of the lipid species with one double bond more would only be partially separated from the monoisotopic peak with one double bond less and additionally the accurate mass of the latter shifts up to −12 ppm down mass, depending on the intensity ratios of both compounds. In such a case a search algorithm capitalizing on the almost nonshifted *M* + 1 isotopologue of the species with one double bond less can still identify and subsequently quantify both overlapping species. Furthermore, it has to be mentioned that also other isotopes like ^2^H, ^15^N, or ^18^O could potentially have an impact on quantitation if they are neither mass resolved nor isotopically corrected. But since ^2^H has a natural abundance of just 0.015% and the number of N or O atoms in lipids rarely exceeds 3 or 17, respectively, their contribution to *M* + 1 and *M* + 2 peaks is in most lipids negligible. For very accurate quantitation of minute amounts of a compound in the presence of large potentially overlapping isotopic peaks in the same spectrum it is in any event advisable to use a resolution in excess of 500,000 for fine isotopic resolution. Another example for isobaric species would be overlapping protonated and sodiated adducts as exemplified in Table 1 for PC 34:1 and PC 36:4 (^12^C_2_ vs. ^1^H_1_^23^Na_1_), which already needs a resolving power of around 600,000, but can be avoided by selective suppression of sodiated adducts by addition of ammonia salts (Brugger et al., 1997). Bielow et al. (2017) show in a very systematic manner the various isotopic patterns to be encountered in lipidomics and the mass‐dependent mass resolution needed for resolving certain isobars. Although the high mass resolution is by itself not able to resolve all the isomeric possibilities arising by the sheer combinatorial power of the various esterified fatty acyls, it is nevertheless a very helpful instrumental asset for reducing the number of lipid candidates and even more so for increasing the certainty of analysis by contributing high confidence elemental compositions. When for example all possible molecular lipid species at nominal mass 773 are calculated by taking into account the fatty acids mentioned at The Lipid Web (https://lipidhome.co.uk/), just for PC we end up with 202 possibilities (Fig. 2). But one has to keep in mind that this number still does not reflect any branched, cyclic, oxygenated, or in other ways modified rarely occurring fatty acids, which would increase this number even more. The most important advantage of high mass resolution in this example is the separation of diacyl and ether lipids, which differ by one oxygen in their sum composition (C_44_H_88_O_7_N_1_P_1_ vs. C_43_H_84_O_8_N_1_P_1_) and of highly unsaturated even carbon numbered fatty acyl PC species from monounsaturated odd carbon numbered fatty acyl PC species (C_44_H_72_O_8_N_1_P_1_ vs. C_43_H_84_O_8_N_1_P_1_). In such a case a mass resolution of around 45,000 will be sufficient to cut the number of possibilities from 202 down to 58. At this point of structure elucidation, high mass resolution of intact lipid molecules nevertheless runs into its limits, because the remaining 58 possibilities are all isomers with exactly the same elemental composition and can only be separated by fragmentation, chromatography or ion mobility.

**Table 1:** Examples for various isobaric and isomeric overlaps commonly encountered in lipidomics including their mass difference and the roughly required mass resolution for separating them

Lipid species	Adduct	Isotope	Mass [*m*/*z*]	Elemental composition	Δm [*m*/*z*]	R (FWHH)
PC 36:0	H^+^	monoisotopic	790.631987	C_44_H_89_O_8_N_1_P_1_	0.0939	20,000
PE 40:7	H^+^	monoisotopic	790.538087	C_45_H_77_O_8_N_1_P_1_		
PC 38:4	H^+^	monoisotopic	810.600687	C_46_H_85_O_8_N_1_P_1_	0.07278	30,000
PS 38:5	H^+^	monoisotopic	810.527907	C_44_H_77_O_10_N_1_P_1_		
SM d34:1	H^+^	*M* + 1	704.578167	C_38_^13^C_1_H_80_O_6_N_2_P_1_	0.05573	30,000
PC 30:1	H^+^	monoisotopic	704.522437	C_38_H_75_O_8_N_1_P_1_		
PC 33:1	H^+^	monoisotopic	746.569387	C_41_H_81_O_8_N_1_P_1_	0.03639	45,000
PC a34:1	H^+^	monoisotopic	746.605777	C_42_H_85_O_7_N_1_P_1_		
DG 36:0	NH_4_^+^	monoisotopic	642.603071	C_39_H_80_O_5_N_1_	0.01527	90,000
CE 16:0	NH_4_^+^	monoisotopic	642.618341	C_43_H_80_O_2_N_1_		
PC 34:1	H^+^	*M* + 2	762.591747	C_40_^13^C_2_H_83_O_8_N_1_P_1_	0.00894	180,000
PC 34:0	H^+^	monoisotopic	762.600687	C_42_H_85_O_8_N_1_P_1_		
PC 36:4	H^+^	monoisotopic	782.569387	C_44_H_81_O_8_N_1_P_1_	0.002406	600,000
PC 34:1	Na^+^	monoisotopic	782.566981	C_42_H_82_O_8_N_1_P_1_Na_1_		
PC 33:1	H^+^	monoisotopic	746.569387	C_41_H_81_O_8_N_1_P_1_	0	–
PE 36:1	H^+^	monoisotopic	746.569387	C_41_H_81_O_8_N_1_P_1_		

## III INSTRUMENTAL PLATFORMS

### A Sector Mass Spectrometry

From a historical perspective sector mass spectrometers were among the first instruments available for high mass resolution lipid analysis and were used in this field from the early 80s on (Jensen & Gross, 1987). The particular merits of sector instruments are not only substantiated by their high mass resolution and mass accuracy but also by the availability of high energy collisional‐activated dissociation (CAD) resulting in charge remote fragmentation (CRF) reactions, which allow for localization of structural details such as double bonds, branches, epoxy‐, hydroxy‐, cyclopropane, and cyclopentane moieties (Jensen, Tomer, & Gross, 1985, 1986; Tomer, Crow, & Gross, 1983; Tomer, Gross, & Deinzer, 1986). The underlying mechanism of fragmentation is a highly specific 1,4‐elimination of H_2_ which results in the loss of methane, ethane, propane, etc. from the omega terminus of fatty acyls. These neutral losses have a very predictable pattern as long as fatty acyls are straight‐chained, saturated and without any other substituents. But whenever such “obstacles for fragmentation” occur in a fatty acyl moiety the fragmentation pattern starts to change distinctively, thus indicating the position and nature of irregularities in the homologous carbon chain (Tomer, Crow, & Gross, 1983; Jensen et al., 1985). Furthermore, it was proven that it was even possible to determine the double bond locations in fatty acyls esterified in triacylglycerols (TG) by CRF (Cheng, Pittenauer, & Gross, 1998). Therefore CRFs are still up to today a powerful tool for in‐depth structural elucidation of lipids.

### B Matrix‐Assisted Laser Desorption Ionization‐Time‐of‐Flight (MALDI‐TOF)

MALDI‐TOF instruments are in use for analysis of lipids since the late nineties (Schiller et al., 1999), but although these instruments are able to quickly deliver data when the right matrix is found (Leopold et al., 2018), their usage is still rather limited. This might be attributed to some limitations inherent to MALDI‐TOF technology: MALDI is not easily coupled with chromatography and thus lacks pre‐separation, it does not have any precursor selection for reliable fragment spectra unless MALDI‐TOF/TOF is used and it also lacks the resolution of Q‐TOF, Orbitrap, and FT‐ICR‐MS instrumentation. Therefore, when the matrix is optimized, MALDI‐TOF is rather used as a fast screening method with low identification confidence. This is very well exemplified by the fast acquisition of differential lipid profiles on urine, which serve as a starting point for further in‐depth exploration of lipids showing a significant difference between statistical groups (Tipthara & Thongboonkerd, 2016). Another niche of application for this technology is the use of MALDI‐TOF/TOF for in‐depth structural characterization of lipids, which capitalizes on the availability of high energy CAD spectra in these instruments. This results in CRF patterns similar to sector mass spectrometry, which allow the allocation of fatty acid *sn*‐positons, double bonds and other modifications at the fatty acyl tails of lipids (Pittenauer & Allmaier, 2009), although the isolation window of four *m*/*z* for MS/MS generation can become a so far unresolved challenge when working on lipids. The drawback of MALDI‐TOF/TOF for the structure elucidation of lipids is its current lack of automatization and the missing embedding into high throughput lipidomic workflows.

### C Mass Spectrometry Imaging

The eventually most important application of MALDI‐TOF these days is mass spectrometry imaging. This is performed by placing a few micrometer thick cryo‐dissections of organs onto a MALDI target, covering them with MALDI matrix and subsequently scanning them in two dimensions by the laser in pixels of a few micrometers (Wang, Wang, & Han, 2018). The resulting mass spectra can be reconstructed to give a two‐dimensional picture of *m*/*z* values, which eventually allow location of certain lipids in the respective tissue. Recently, Ellis et al. (2018) showed on an LTQ‐Orbitrap instrument the potential of coupling between high‐resolution shotgun lipidomics and MALDI imaging. At a pixel size of 40 µm one FT‐MS full scan at a resolution of 240,000 and parallel low‐resolution IT‐MS/MS scans in data‐dependent acquisition (DDA) mode were acquired. Both scan types were merged by the software and each pixel was processed like one sample of a shotgun experiment. This finally led to two‐dimensional rat cerebellum images at a lateral resolution of 40 µm where lipid assignment from high‐resolution full scan spectra was further corroborated by characteristic fragments from the respective MS/MS spectra. In a similar manner, distribution of sulfoglycosphingolipids in tumor tissue was determined by MALDI imaging on an LTQ‐Orbitrap mass spectrometer, taking into account high mass resolution FT‐MS full scans and MS/MS scans by CAD, pulsed Q collisional dissociation (PQD), and higher energy collision activated dissociation (HCD) (Jirasko et al., 2017). Another interesting approach for pinpointing spatial distribution of lipids is laser capture microdissection of tissue slices with subsequent lipid extraction and shotgun lipidomics (Knittelfelder et al., 2018). The big advantage of this method is the increased amount of time which can be spent on each pixel allowing for various targeted selected ion monitoring (t‐SIM) and MS/MS experiments and results in a very deep coverage of each pixels lipidome. When a lateral resolution beyond 1 µm is needed, then TOF‐SIMS or SIMS‐FT‐ICR‐MS would be the instrumentation of choice (Smith et al., 2013; Desbenoit et al., 2014). Besides a spatial resolution down to 100 nm, which basically already enables coarse subcellular localization of lipids, the second big advantage of SIMS is that it is a matrix‐free method, thus excluding all sources of error arising from matrix deposition. On the downside, SIMS is prone to produce in‐source fragmentation, eventually resulting in loss of information on product‐precursor relationships.

### D Shotgun Lipidomics

The term shotgun lipidomics comprises a variety of different instrumental platforms operated in direct infusion and mostly relying on electrospray ionization (ESI). Due to the lack of any chromatographic separation, high mass resolution is increasing the confidence of analysis enormously in such a setting, even though shotgun approaches literally always also have to rely on fragmentation of intact lipid ions in a further MS/MS step. While in the pioneering phase of lipidomics in the 90s most instrumental platforms were triple quadrupoles operated under nominal mass resolution (Han & Gross, 1994, 2005; Brugger et al., 1997; Liebisch et al., 1999, 2002), the development in the last two decades clearly shifted shotgun lipidomics toward high mass resolution equipment, consisting particularly of Q‐TOF and Orbitrap instrumentation (Ekroos et al., 2002; Schuhmann et al., 2006, 2011, 2012; Ejsing et al., 2009; Almeida et al., 2015; Ellis et al., 2018; Horing et al., 2019). On the infusion side of such platforms the Nanomate nanoESI chip from Advion Inc. can be regarded as a very useful complementary piece of equipment, because it uses one nanoESI spray needle for each sample and thus minimizes carry over effects which are frequently observed when using just syringe infusion (Schwudke et al., 2006). Furthermore, nanoESI increases signal intensities and diminishes the amount of sample needed per injection (Hsu, 2018). Generally, the biggest advantage of shotgun lipidomics over LC‐MS lipidomics is the quantitative aspect. Because of its stable ionization environment any fluctuations arising from chromatography, like changing mobile phase composition, matrix or target compound concentration can be excluded (Han & Gross, 2005; Schwudke et al., 2006; Horing et al., 2019). Thus, only one internal standard per polar lipid class is usually sufficient, because the ionization efficiency depends just on the polar head group where the charge is located and not on the varying fatty acyl chains (Wang, Wang, & Han, 2017). Regarding robustness, an interesting shotgun lipidomics study showed a very good stability of lipid concentrations in human plasma over a range of 3.5 years with coefficients of variation mostly below 15%, which would qualify this method even for U.S. Food and Drug Administration studies according to good laboratory practice (Heiskanen et al., 2013). The drawback of shotgun lipidomics are its inherent ion suppression effects, because all lipids are ionized together without any pre‐separation. This can in the worst case lead to complete suppression of minor constituents of the lipidome, especially when they have to be detected simultaneously beside highly abundant other compounds. By use of intrasource separation, ion suppression effects can be alleviated for certain lipid classes, resulting in specific ionization enhancement of certain lipid classes (Han et al., 2006). Furthermore, a recently published concept to at least partially deal with this issue is spectral stitching (Southam et al., 2016; Schuhmann et al., 2017). The proposed workflow parses the range of a full scan MS^1^ spectrum into certain extremely wide selected ion monitoring (SIM) ranges of 20–50 *m*/*z* units, which are acquired in a sequential manner. These SIM spectra are subsequently stitched together by the software and result in one single full scan spectrum at the end of this process. This circumvents at least the ion suppression effects arising from limited fill capacities of ion storing devices such as Orbitrap or ICR cells. But it nevertheless leaves the ion suppression effects in the ESI source untouched. A particular shortcoming of shotgun lipidomics, when compared with chromatography based approaches, is the inability to separate isomeric lipid species just by mass. Although this can be solved by fragment spectra, an additional chromatographic dimension would provide a higher degree of certainty in such cases. But as eluded in the previous chapter even some isobaric lipid overlaps can become a challenge when Q‐TOF instead of Orbitrap or FT‐ICR‐MS technology is used. When using instrumentation with a resolution of 500,000 or even above isotopic labeling experiments are an interesting application for determination of metabolic fluxes by using isotopes such as ^15^N or ^17^O (He et al., 2011). These isotopes have a very low natural abundance which has been shown to be highly beneficial for ^15^N labeling in HepG2 cells (Schuhmann et al., 2017). The advantage of Q‐TOF mass spectrometry is its acquisition speed, which allows for data‐independent acquisition (DIA) MS/MS^ALL^ methods as recently proposed by Gao et al. (2018). Due to the sheer acquisition speed of the TripleTOF used, this workflow is able to automatically acquire MS/MS spectra with a precursor selection window of 1 Da for a mass range as wide as 1000 Da, which has the advantage of 100% MS/MS spectra coverage for the whole mass range scanned. The nominal mass parsing of the scan range also circumvents the drawback of previous MS/MS^ALL^ concepts, which operated with wider isolation windows and thus could only compensate the loss of unambiguous precursor–fragment relationships by additional use of chromatography and retrospective *in silico* retention time‐fragment relationship alignment. A further step of improvement of MS/MS^ALL^ technology termed MS^ALL^ was performed on an Orbitrap Fusion Tribrid and fully capitalizes on the wealth of fragmentation options available on this type of instrument (Almeida et al., 2015). This method also includes full scan spectra at a resolution of 450,000 (*m*/*z* 200) in positive and negative polarity in a low and high *m*/*z* range. MS/MS spectra were acquired in 1.0008 Da steps over the entire *m*/*z* range in the HCD cell and in the linear ion trap, each at a resolution of 30,000. Additionally, MS^3^ spectra on selected lipids were acquired in the linear ion trap. The only shortcoming of this method could turn out to be the collision energy settings, which are eventually not completely optimal for each lipid class, particularly when a huge number of different lipid classes is to be analyzed.

Recently, Ellis et al. (2018) showed on an LTQ‐Orbitrap instrument the potential of coupling between high‐resolution shotgun lipidomics and MALDI imaging. At a pixel size of 40 µm one FT‐MS full scan at a resolution of 240,000 and parallel low‐resolution IT‐MS/MS scans in DDA mode were acquired. Both scan types were merged by the software and each pixel was processed like one sample of a shotgun experiment. This finally led to two‐dimensional rat cerebellum images at a lateral resolution of 40 µm where lipid assignment from high‐resolution full scan spectra was further corroborated by characteristic fragments from the respective MS/MS spectra. In a similar manner, the distribution of sulfoglycosphingolipids in tumor tissue was determined by MALDI imaging on an LTQ‐Orbitrap mass spectrometer, taking into account high mass resolution FT‐MS full scans and MS/MS scans by CAD, PQD, and HCD (Jirasko et al., 2017). Another interesting approach for pinpointing spatial distribution of lipids is laser capture microdissection of tissue slices with subsequent lipid extraction and shotgun lipidomics (Knittelfelder et al., 2018). The big advantage of this method is the increased amount of time that can be spent on each pixel allowing for various t‐SIM and MS/MS experiments and results in a very deep coverage of each pixels lipidome.

If deeper structural elucidation of lipids including localization of fatty acyl double bond positions is of interest, UV‐induced photodissociation (UVPD) might in future become the fragmentation technique of choice. In a nutshell, activation of bond cleavages between allylic methylene groups and the corresponding double bond by a 193 nm UV laser is the mechanism, by which unambiguous double bond localization in fatty acyls of phospholipids and long‐chain bases of sphingolipids has been proven on Orbitrap instrumentation recently (Ryan et al., 2017; Williams et al., 2017). A further method for double bond localization and separation of regioisomers would be OzID, which relies on the recation of ozone with aliphatic double bonds, similarly to mechanisms of lipid peroxidation (Brown, Mitchell, & Blanksby, 2011). This reaction results via the generation of ozonides and Criegee intermediates in generation of truncated aldehydes and Criegee ions, with the site of truncation indicative for the double bond location. The drawback of OzID are its instrumental demands, because the mass spectrometer has to be customized for getting ozone into the collision cell or ion trap. Recently, the UV‐induced Paterno–Büchi reaction of aliphatic double bonds with acetone came into the focus of lipidomics, because it enables localization of double bonds by analysis of its reaction products, which are consistently truncated at the positions of fatty acyl double bonds (Zhang et al., 2019). When acetone is added post‐column and an UV emitter is placed in front of the ion source, this online reactor is even able to be coupled with LC‐MS instrumentation.

### E LC‐MS

**Figure 3:**
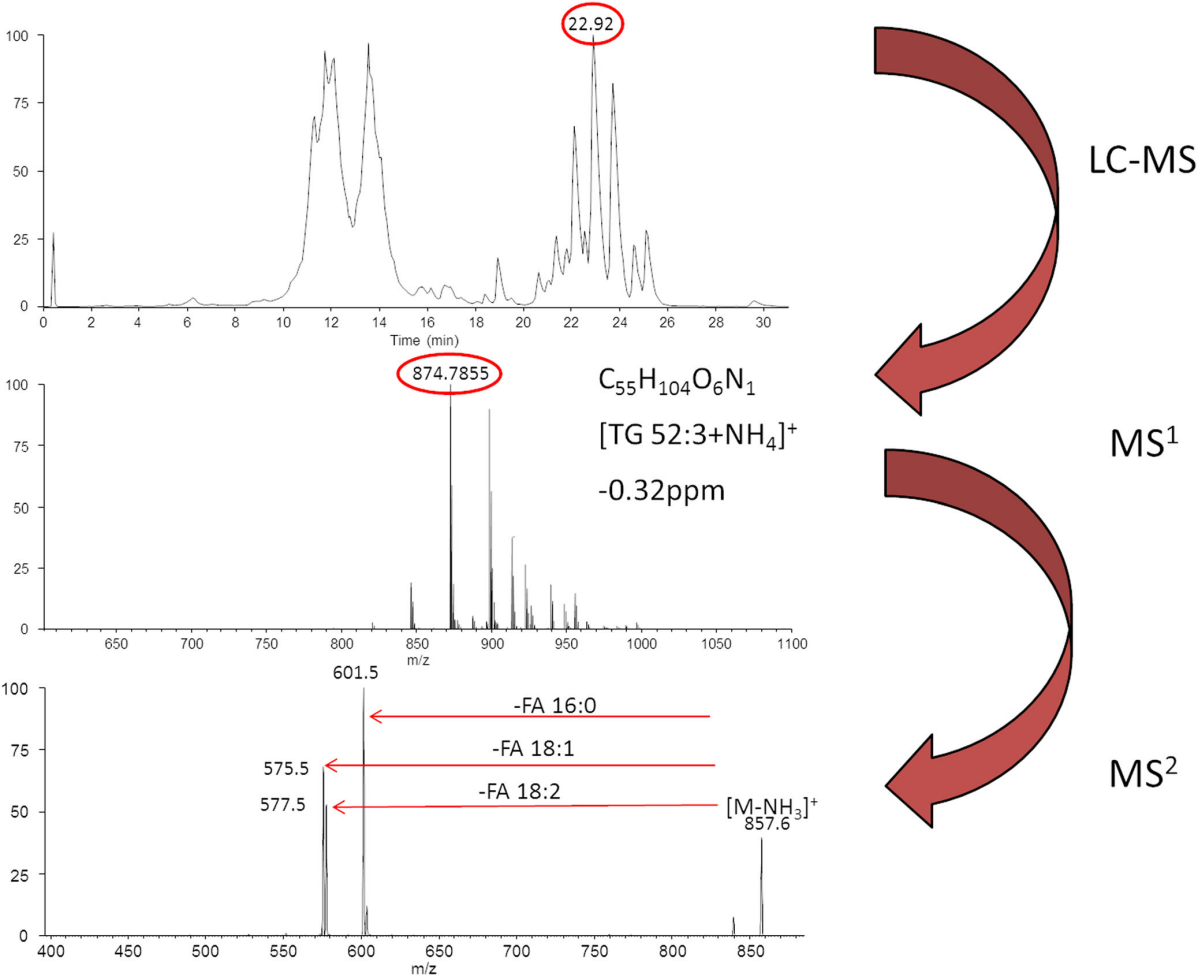
Workflow of a high‐resolution liquid chromatography–mass spectrometry (LC‐MS) lipidomics platform. The upper panel shows the total ion chromatogram of a mouse liver extract acquired in positive electrospray ionization mode at a mass range *m*/*z* 350–*m*/*z* 1100. The middle panel shows the spectrum at retention time 22.92 min, containing the [TG 52:3 + NH_4_]^+^ ion at *m*/*z* 874.7855 with a mass accuracy of −0.32 ppm. The lower panel shows the corresponding MS/MS spectrum of *m*/*z* 874.7855 acquired in a linear ion trap in data‐dependent acquisition mode. Depicted are the confirmative fatty acid‐neutral losses of TG 52:3. [Color figure can be viewed at wileyonlinelibrary.com]

The two most widely used approaches in LC‐MS are reversed‐phase chromatography and hydrophilic interaction liquid chromatography (HILIC) (Holcapek, Liebisch, & Ekroos, 2018). While reversed‐phase chromatography separates lipids by composition of their fatty acyl chains, HILIC separates lipids according to their polar head groups, which results indistinct lipid class separation. The fundamental separation mechanism in reversed‐phase chromatography of lipids is described by the equivalent carbon number model predicting increasing retention times with an increasing fatty acyl carbon number and decreasing retention times with an increasing number of double bonds. Therefore it is possible to separate lipid species from the same lipid class by their cumulative carbon number‐double bond index and with increasing chromatographic plate number it is even possible to separate isomeric species according to their fatty acyl composition (Knittelfelder et al., 2014). Due to this advantage of lipid molecular species separation, many LC‐MS lipidomics platforms are based on reversed‐phase chromatography coupled to Orbitrap, FT‐ICR‐MS, or Q‐TOF instruments (Hein et al., 2009; Fauland et al., 2011; Knittelfelder et al., 2014; Sala et al., 2015; Triebl et al., 2017; Williams et al., 2017; Griffiths et al., 2018; Holcapek, Liebisch, & Ekroos, 2018; Schott et al., 2018; Schlotterbeck et al., 2019). However, it has to be mentioned that carryover effects can become a problem in reversed‐phase chromatography, particularly when C18 or even C30 columns are used (authors unpublished observations). Thus it is important to closely monitor any carry‐over effects by running solvent blanks every few (e.g., 10) samples and allowing several minutes of washing and equilibration time. Figure 3 exemplifies the merits of chromatographic separation coupled to high‐resolution mass spectrometry: The upper panel shows reversed‐phase chromatographic separation of a mouse liver lipid extract in a total ion chromatogram according to fatty acyl composition and lipid class, but the chromatographic peak at 22.92 min still contains many chromatographically overlapping TG species. Nevertheless the high mass resolution of an Orbitrap instrument is able to separate the various adduct ions and their isotopic peaks at the given retention time and subsequently identifies the mass at 874.7855 as an elemental composition potentially corresponding to a [TG 52:3 + NH_4_]^+^ ion. In parallel the linear ion trap acquires an MS/MS spectrum of this mass peak in DDA mode, which firstly corroborates the identity of TG 52:3 and secondly elucidates it to be an TG 16:0_18:1_18:2 by the corresponding fatty acyl neutral losses of molecular weight of 256, 282, and 280. While such a setup often shows very high selectivity relying on retention time, exact mass of intact lipids and characteristic MS/MS fragments, the quantitative aspects are its biggest disadvantage. In contrast to shotgun lipidomics or HILIC it is not sufficient to use just one or two internal standards per co‐eluting lipid class, but ideally one stable isotope‐labeled internal standard per compound, because with changing matrix and mobile phase composition also ion suppression effects change from spectrum to spectrum. Since one internal standard for each lipid species is for economic reasons usually not feasible, four to ten internal standards per lipid class distributed over its retention time range are a good compromise to achieve at least semi‐quantitative data (Triebl et al., 2017). Another interesting recently proposed approach is called lipidome isotope‐labelling of yeast (LILY) and relies on a fully ^13^C‐labeled yeast lipidome from *pichia pastoris* grown on completely ^13^C‐labeled cell culture medium (Rampler et al., 2018). This concept results in availability of one stable isotope‐labeled internal standard for each lipid species as long as the same organism is used. Nevertheless, all the naturally grown ^13^C‐labeled lipids from this yeast extract need in first place to be quantified by known amounts of nonlabeled reference standards, therefore shifting the bottleneck of standardization from the availability of isotope‐labeled internal standards to the availability nonlabeled reference compounds. Owing to their separation power, reversed‐phase chromatography based lipidomics platforms are often used in DDA mode either for targeted or for nontargeted lipidomics. Good examples for targeted analysis with high‐resolution instruments would be lipid class‐specific methods focused on sphingolipids or sterols on a Q‐Exactive in parallel reaction monitoring (PRM) mode (Peng et al., 2017; Schott et al., 2018). When used for nontargeted analysis high mass resolution is even more imperative because in such a setting it might become important to determine the identity of so far unknown lipid structures, which is close to impossible without the availability of accurate mass on molecular adduct ions and fragment ions alike. In a comparative nontargeted lipidomics study including 7 Q‐TOF models, one Q‐Exactive and one TOF instrument, it was shown that the results were quite similar independently of the high‐resolution machinery used (Cajka, Smilowitz, & Fiehn, 2017). Although the general merit of nontargeted omics approaches is the reduction of complexity because ten thousands of features are reduced to eventually just a few hundred significantly regulated features, it is nevertheless a tedious and daunting job to unambiguously identify all the corresponding lipids (Triebl et al., 2017). It is needless to say that in such a process high mass accuracy is absolutely mandatory and improves the certainty of lipids identified by a great deal. Just when taking into account C, H, O, N, P, and S in a distribution typical for lipids (no more than 18 O, 3 N, 2 P, and 2 S) including its most abundant isotopes (^13^C and ^34^S), assuming just even electron ions formed by ESI and no more than eight ring double bond equivalents, results at *m*/*z* 810.60073 ([M + H]^+^ of PC 38:4) in 11 possible elemental compositions at 1 ppm mass accuracy and 48 possible elemental compositions at 5 ppm mass accuracy.

When quantitation of lipids is needed, HILIC has a clear advantage in comparison with reversed‐phase chromatography. Since all lipids from a certain lipid class are eluting in a narrow retention time range, each lipid class can almost be regarded as one chromatographic peak with very similar mobile phase composition and matrix effects. Consequently the response factors for individual molecular lipid species within the same lipid class are very close to each other and it is possible to obtain good quantitative results with just one or eventually two internal standards, similarly to shotgun lipidomics (Cifkova et al., 2012). Therefore HILIC separation coupled to high‐resolution mass spectrometry is a combination worth considering and starts to gain more attention recently (Triebl et al., 2014; Hajek et al., 2017).

Another recent development in lipidomics is the use of nanoHPLC, which was shown to tremendously increase the coverage of detected lipids. While conventional narrow‐bore reversed‐phase HPLC could separate 127 molecular lipid species reversed‐phase nano HPLC could separate 436 molecular lipid species, which were subsequently identified on a Q‐Exactive (Danne‐Rasche, Coman, Coman, 2018). These results could potentially pave the road toward a much wider use of nano HPLC systems in lipidomics, if the robustness issues typically arising from miniaturization of chromatography can be overcome.

02wOver the last decade, supercritical fluid chromatography (SFC) has come to a stage of maturity in lipidomics, at which application is conceivable routinely. The big advantage of SFC over conventional HPLC is better chromatographic separation at shorter elution times. The compatibility of supercritical carbon dioxide as mobile phase with ESI is ensured by addition of a makeup liquid between column and ion source. Thus it has become possible to separate as many as 305 lipid species from 25 lipid classes in a chromatographic run of just 6 min by ultra‐high‐performance SFC (UHSFC) (Lisa & Holcapek, 2015). In a comparison of UHSFC with UHPLC it was shown that UHSFC could identify by a factor of 3.4 more lipids in 40% less run time when coupled to a Q‐TOF (Lisa et al., 2017).

## IV DATA PROCESSING

### A Shotgun Software Tools

The challenges in the field of shotgun lipidomics for data processing are on one hand the simultaneous ionization of all components of a sample and on the other hand the acquisition of samples with multiple strategies, for example, ionization in positive and negative mode or extractions with different chemical and/or physical conditions to improve ionization efficiency for different lipid classes (Han et al., 2004; Jiang et al., 2007). In order to process this conglomerate of collected data sets, various specialized software tools are available to process these samples. The automated multidimensional mass spectrometry‐based shotgun lipidomics is a building‐block concept with a combination of a nontargeted and a targeted approach to identify and quantify data from several shotgun lipidomics experiments. This concept of feature identification is based on information of the total number of carbon atoms, the number of double bonds, the chemical formulas, the monoisotopic mass, and building blocks, for example, chain, backbone, and head groups which in combination represent the whole lipid (Yang et al., 2009). LipidXplorer software is based on declarative molecular fragmentation query language to identify and quantify obtained spectra on an individually defined identification routine (Herzog, Schwudke, & Shevchenko, 2013). It is a highly adaptable device‐independent system, which can handle low‐resolution data, precursor and neutral loss scans (Herzog et al., 2012) as well as bottom‐up (Schuhmann et al., 2011) and top‐down (Schwudke et al., 2007) approaches. Further typical shotgun lipidomics tools are LipidView/LipidProfiler from AB SCIEX (Ejsing et al., 2006), LipidInspector (Schwudke et al., 2006), and The analysis of lipid experiments (ALEX). ALEX is a graphical user interface (GUI) based framework consisting of 6 modules and is designed to process high‐resolution data from multiplex shotgun workflows from raw data conversion to final lipid quantification. The lipid annotation is based on a database with stored information on 85 lipid classes and over 20,000 lipid species (Husen et al., 2013).

### B LC‐MS Software Tools

In contrast to direct infusion mass spectrometry, raw data from LC‐MS methods cannot be exported as averaged profile data for further processing. Each data point may belong to another representative feature. This illustrates a fundamental difference between the requirements for data processing packages and tools of shotgun‐MS and LC‐MS approaches. Two main acquisition techniques are widely used: DDA and DIA. DIA can be subdivided into sequential window acquisition of all theoretical fragment‐ion spectra (SWATH), where additional isolation windows from 20 up to 50 Da are required to simplify MS^1^ spectra connection, and all‐ion fragmentation (AIF), MS^ALL^ respectively MS^E^ (Fenaille et al., 2017). With the DDA approach, data are recorded as full scan spectra at MS^1^ level and MS/MS spectra are automatically generated based on their intensity and/or external precursor lists. This clear relationship between precursor and fragment ions is beneficial compared with DIA approaches, with the limitation that minor contaminations caused by a 1 Da precursor selection window and co‐eluting isomeric features are possible. Another DDA disadvantage is the lack of MS/MS confirmation spectra of all precursor ions of interest. An experiment with a standard mixture of 40 metabolites showed 85% MS/MS coverage (Benton et al., 2015), however, this can depend heavily on the sample matrix and chromatography. This problem primarily affects low‐intensity ions and retention times similar to target ions with high intensity. In MS^E^ and SWATH approaches all features are fragmented which theoretically means 100% MS/MS coverage. This results in highly complex MS/MS spectra, where proper software processing tools are required. Several software solutions are available for DDA and DIA as shown in Table 2.

**Table 2:** Overview of the main features of selected LC‐MS‐based lipidomics software tools

Name	Acquisation mode	Raw file conversion	Data processing	Filtering options	MS/MS annotation	Normalize to lipid IS	Licence	Literature
XCMS‐Family	DDA	External	Centwave, OBI‐warp	Adducts, RT, Isotope, Polarity	List, or similarity search DB (*m*/*z*)	Yes	General Public License version 2.0	Mahieu, Genenbacher, and Patti (2016), Benton et al. (2008)
Open‐MS	DDA and DIA	External	FeatureFinderMetabo	Adducsts, RT, Isotope	Spectral matching	Yes	Three‐clause BSD license	Pfeuffer et al. (2017)
LipidSearch v5.0	DDA	Built‐in	–	Adducts, RT, Isotope, polarity	Rule (*m*/*z* and int)	Yes	Commercial	Breitkopf et al. (2017)
MS‐DIAL 4.0	DDA, DIA, and IM	External	Linear‐weighted moving average	Adducts, RT, Isotope	Modified Dot Prod (*m*/*z* and int)	Yes	“Open Source”	Tsugawa et al. (2015)
LipidMatch Flow Normalizer	DDA and DIA	Built‐in	Internal (MZmine2)	Adducts, Polarity, RT, blank filtering	Rule (*m*/*z*)	Yes	Creative Commons Attribution 4.0	Koelmel et al. (2017, 2019)
LDA2	DDA	Built‐in	3D algorithm	Isotope, RT	Rule (*m*/*z* and int)	Yes	GNU General Public License v3.0	Hartler et al. (2011, 2017)
Liquid	DDA	Built‐in	Built‐in	No/External	Log‐likelihood scores (fragment)	(Yes)	Apache License, Version 2.0	Kyle et al. (2017)
SimLipid	DDA/DIA	Built‐in	–	Adducts, Polarity, RT	–	Yes	Commercial	–
GREAZY/Lipidlama	DDA	Built‐in	Bins, scoring (HGD) (*m*/*z*, int), FDR	No/External	Baysian (*m*/*z*)	No	“Open Source”	Kochen et al. (2016)
MZmine2	DDA	Built‐in	Exact Mass, several	Adducsts, RT, Isotope	Limited/External	Yes	General Public License v2.0	Pluskal et al. (2010)
LipiDex	DDA	External	External	Adducts, RT, Polarity	Modified Dot Prod (*m*/*z* and int)	No	MIT License	Hutchins, Russell, and Coon (2018, 2019)
LipidBlast	DDA	External	External	No/External	Modified Dot Prod (*m*/*z* and int)	No	Creative‐Commons By‐Attribution	Kind et al. (2013, 2014)

There is a group of software tools which only specialize in one or two steps. In combination, however, they are very flexible and can cover the entire workflow:

(i) raw file conversion, for example, msConvert (Adusumilli & Mallick, 2017) or Reifycs Abf converter. (ii) For peak picking, blank filtering, adduct, and polarity combining, and isotope filtering typical solutions are omics software tool name (XCMS) (Mahieu, Genenbacher, & Patti, 2016) in combination with Camera (Mahieu, Genenbacher, & Patti, 2016) or MZmine 2 (Pluskal et al., 2010). (iii) Several specialized software tools are available for MS/MS annotation XCMS2 (Benton et al., 2008), LipiDex (Hutchins, Russell, & Coon, 2018, 2019), LipidBlast (Cajka & Fiehn, 2017), or LipidIMMS (Zhou et al., 2019), a package solution for annotation with an additional ion mobility dimension. The advantage of package‐based workflows is that they can be customized for each device and research area, but setting them up can be more complicated and time‐consuming. To simplify data processing, there are workflow‐oriented software solutions such as LipidMatch Flow, in which msConvert for the manufacturer‐specific raw file conversion and MZmine 2 for data processing and filtering are integrated in the GUI. There are also some commercial software solutions for the whole workflow like SimLipid (PREMIER Biosoft) or LipidSearch (Thermo Fisher Scientific) and several open‐source solutions like lipid data analyzer 2 (LDA2) (Hartler et al., 2011, 2017), liquid (Kyle et al., 2017), MS‐DIAL 4.0 (Tsugawa et al., 2015), Open‐MS (Pfeuffer et al., 2017), and Greazy (Kochen et al., 2016). The main differences between these software solutions are peak picking and filtering, in which chromatographic data are translated into feature tables and MS/MS features are identified and linked. The different algorithms are listed in Table 2. The main problems are usually over or under annotation. The quality strongly depends on the complexity of the measured samples, the ionizability of the ions, the compound concentration and the combination of the MS device and optimized algorithm parameters.

MS/MS annotation in lipidomics is the main difference to other areas of MS such as metabolomics or proteomics. Due to the chemical structure (polar head group and acyl chain), they can be annotated with databases on fragmentation ion similarity scoring and/or according to structure specific fragmentation rules. MS‐Dial, LipiDex, and LipidBlast MS/MS annotation are based on similarity, for example, LipiDex is using a modified dot product to score experimental MS/MS data to MassBank (Horai et al., 2010), LipidBlast (Kind et al., 2013), and NIST 12 MS/MS library. LDA 2 uses rule decisions based on text files to process MS/MS data. The rules can be easily extended based on diagnostic ions, neutral losses, intensity ratios, exclusions based on false‐positive ions and also combinatory rules (Hartler et al., 2017). There is a group of online tools and software packages that specialize in computational approaches to compound annotation. In silico fragmentation, the software identifies unknown compounds by comparing and ranking theoretical MS/MS spectra with experimental MS/MS spectra. MetFrag 2.2 (Ruttkies et al., 2016) is a web service that can be used as a desktop version or integrated into the XCMS and OpenMS workflow. It supports structure imports from common databases such as PubChem, KEGG (Kanehisa et al., 2008; Kanehisa & Sato, 2019), ChemSpider and user‐defined data. Another software tool is Competitive Fragmentation Modeling–ID (CFM‐ID) 3.0 (Allen et al., 2014; Djoumbou‐Feunang et al., 2019), which contains a compound library obtained from the METLINE metabolite database with different collision energies for fragmentation evaluation. It also has a rule‐based library designed specifically for larger molecules like lipids to speed up prediction and improve accuracy. Despite the different software solutions, the exact structure identification is still a difficult task, even if the quality of annotation has been massively improved with high‐resolution MS‐devices and MS/MS information. There are few points that are not solved in standard lipidomics approaches, for example, stereoisomers, sn1, sn2, enantiomers or double bond positions.

### C Tools for Batch Normalization

Experiments with a larger number of samples can be challenging because of changing conditions during analysis, such as drift of instrument sensitivity, changes of eluent composition over time, temperature changes and batch interruption due to instrument errors. These factors might lead to lower statistical power (Xiao et al., 2014). It should be noted that data processing and normalization can have a major impact on your results. Therefore, the results should always be checked for plausibility.

There are several data‐driven normalization methods. Li et al. (2016) compared 16 data‐driven normalization methods with four different data sets using the online tool Metapre and categorized them in superior, good, and poor performing methods (Li et al., 2016). Another tool for data‐driven normalization is Metabox (Wanichthanarak et al., 2017). A critical point in data‐driven normalization is that differences due to systematic errors and the variability of sample preparation cannot easily be distinguished from phenotypic variations. Quality control (QC)‐based normalization and/or internal standard (IS)‐based normalization strategies is another approach. A QC is usually a pooled sample which is acquired with a certain frequency between samples. Software solutions based on QC approaches are Batch Normalizer (Wang, Kuo, & Tseng, 2013), which corrects batch variability using LOESS regression, the Random Forest‐based online tool SERRF (Fan et al., 2019), the Support Vector Machine based StatTarget (Luan et al., 2018), and EigenMS (Karpievitch et al., 2014). IS‐based normalization works with several standards which are added to each sample. Since the availability of standard compounds is limited and the costs can be very high, at least 1 standard per lipid class should be added. The number of standards required also depends on the method used, for example, reversed‐phase, HILIC or shotgun MS. Best‐Match Internal Standard (B‐MIS) (Boysen et al., 2018) normalizes peak areas based on isotopic‐labeled internal standards which behave similarly during the analysis. Lipid‐match normalizer (Koelmel et al., 2019) is an extension of the Lipidmatch tool, which uses a ranking system to find the most suitable lipid standard for each analyte. LDA 2 (Hartler et al., 2011) follows a similar approach with internal standards and an automatic assignment of the respective standards to the targets. There are also tools which are combining data‐driven approaches with IS‐and/or QC‐based approaches such as NOREVA (Li et al., 2017) (NORmalization and EVAluation of MS‐based metabolomics data). It is an online service with 24 different data‐driven normalization methods with QC‐based or QC/IS‐based normalization strategies and evaluates the performance for multiple testing. Despite these different tools, it is still difficult to compare data between different MS platforms. A strategy based on internal standards or a combined strategy is therefore the most promising way to achieve platform‐independent results

## V REPORTING AND QUALITY STANDARDS

Little more than a decade ago, when lipidomics was still in its very infancy, an international group of acknowledged researchers in the field founded the International Lipids Classification and Nomenclature Committee (ILCNC), which created a logically structured classification system for lipids as depicted in Figure 1 (Fahy et al., 2005). On the basis of this endeavor the LIPID MAPS consortium developed the accompanying LMSD (Sud et al., 2007) and a few years later the LipidomicNet consortium proposed a shorthand nomenclature for reporting of mass spectrometry identified lipids (Liebisch et al., 2013). The core intention of the proposal for a shorthand nomenclature is to report only unambiguous and experimentally proven details according to an annotation system indicative for the analysis depth of uncovered lipid structures. If we stick to the example given in Figure 2, a precursor ion scan *m*/*z* 184 on a triple quadrupole instrument with just direct infusion could indicate a PC species at *m*/*z* 773 but cannot determine if it is a diacyl or an ether species and should therefore be annotated as PC (773). This annotation potentially subsumes already 202 molecular species if just the most commonly detected fatty acid combinations are taken into account. With the availability of a mass resolution of at least 40,000, an ether species could, for example, be experimentally excluded due to the number of oxygens, at least when assuming that the structure under investigation is not an oxidized phospholipid. Thus it could then be labeled PC 35:1, with still 58 possible underlying structures. When now in addition to high mass resolution also MS/MS spectra are available, the mass spectrometrist might be able to infer about the nature of the fatty acyls and eventually even their position. This would, for example, allow annotating the molecule as either PC 17:0_18:1 with unknown fatty acyl *sn*‐positions or as PC 18:1/17:0 when these details are uncovered. At this level the number of structure proposals would be down to 12 or even 6, respectively. Any further elucidation of the remaining structural ambiguities, which basically includes positions and geometries of double bonds, has to involve more sophisticated methods like ozonolysis (OzID), UVPD, or silver ion chromatography (Brown, Mitchell, & Blanksby, 2011; Lisa & Holcapek, 2013; Williams et al., 2017). The legacy of the lipidomics shorthand nomenclature group is the recently founded lipidomics standards initiative, which is an association of 25 leading lipidomics labs for governing the development of standardized practices (https://lipidomics‐standards‐initiative.org/) (Liebisch et al., 2019). The guidelines elaborated by this consortium cover the whole lipidomics workflow from sample collection to data reporting and should in future alleviate collaboration, data exchange and data interpretation in the field. Another recently launched important international initiative is the Plasma Lipidomics Reference Value Group (Burla et al., 2018), which evolved from a recent interlaboratory comparison (Bowden et al., 2017) and has the goal to introduce lipidomics into clinical practice by establishing a panel of diagnostically important lipids including their reference values in plasma. Most recently, the International Lipidomics Society (ILS) emerged from all these activities. ILS is intended as an umbrella organization and communication hub for improved coordination of the many ongoing community efforts and should foster a concerted development of lipidomics as a research field (https://lipidomicssociety.org/).

## VI CONCLUDING REMARKS

Within the last decade lipidomics was one of the fastest‐growing research fields in life sciences and the development of new analytical methods accompanied by the availability of new mass spectrometry equipment had a tremendous impact on this evolution. In this respect, the shift from low toward high‐resolution mass spectrometry is particularly worth mentioning, because this phenomenon runs in parallel to the development of the whole field. Although high mass resolution and the resulting accurate mass are very important ingredients for improving identification certainty of lipids, there are certain natural limitations which cannot be overcome by high‐resolution mass spectrometry alone. Therefore a healthy mix of analytical devices (chromatography, fragmentation techniques, etc.) helps to cope when one is lost in the seemingly overwhelming jungle of lipid isomerism, but high mass resolving power is the natural ally who paves the road for separating these isomers from their isobars in the first place.

## ABBREVIATIONS

HPLC: high‐performance liquid chromatography
LC‐MS: liquid chromatography–mass spectrometry
MALDI: matrix‐assisted laser desorption ionization
Q‐TOF: quadrupole time‐of‐flight
TOF: time‐of‐flight
UHPLC: ultra high‐performance liquid chromatography
